# A Hominin Femur with Archaic Affinities from the Late Pleistocene of Southwest China

**DOI:** 10.1371/journal.pone.0143332

**Published:** 2015-12-17

**Authors:** Darren Curnoe, Xueping Ji, Wu Liu, Zhende Bao, Paul S. C. Taçon, Liang Ren

**Affiliations:** 1 Palaeontology, Geobiology and Earth Archives Research Centre, School of Biological, Earth and Environmental Sciences, University of New South Wales, Sydney, NSW, Australia; 2 Yunnan Institute of Cultural Relics and Archaeology, Kunming, Yunnan, China; 3 Key Laboratory of Vertebrate Evolution and Human Origins, Institute of Vertebrate Paleontology and Paleoanthropology, Chinese Academy of Sciences, Beijing, China; 4 Mengzi Institute of Cultural Relics, Mengzi, Yunnan, China; 5 Place, Evolution and Rock Art Heritage Unit, School of Humanities, Gold Coast Campus, Griffith University, Queensland, Australia; University of Florence, ITALY

## Abstract

The number of Late Pleistocene hominin species and the timing of their extinction are issues receiving renewed attention following genomic evidence for interbreeding between the ancestors of some living humans and archaic taxa. Yet, major gaps in the fossil record and uncertainties surrounding the age of key fossils have meant that these questions remain poorly understood. Here we describe and compare a highly unusual femur from Late Pleistocene sediments at Maludong (Yunnan), Southwest China, recovered along with cranial remains that exhibit a mixture of anatomically modern human and archaic traits. Our studies show that the Maludong femur has affinities to archaic hominins, especially Lower Pleistocene femora. However, the scarcity of later Middle and Late Pleistocene archaic remains in East Asia makes an assessment of systematically relevant character states difficult, warranting caution in assigning the specimen to a species at this time. The Maludong fossil probably samples an archaic population that survived until around 14,000 years ago in the biogeographically complex region of Southwest China.

## Introduction

The number of Late Pleistocene archaic hominin species, their biogeographical distribution, and the timing of their extinction remain important but intractable questions in palaeoanthropology. These issues have been brought into sharp relief over the last half-decade following the accumulation of genomic evidence for interbreeding between the Pleistocene ancestors of living humans and archaic hominins across much of the Old World [1–4]. Yet, in many regions, archaic remains are sparse or absent from the Late Pleistocene fossil record. The few exceptions include: 1) the Neanderthals (*H*. *neanderthalensis*) in Europe, West Asia, and extending at times into southern Siberia, the youngest example being from Mezmaiskaya dating ~39 ka [5]; 2) possibly the North African specimens Dar-es-Soltane and Témara from Morocco [6], with a reported age range of ~110 to 40–20 ka for the associated Aterian lithic industry [7]; 3) fossils from Denisova Cave in southern Siberia consisting of a molar and manual and pedal phalanges that have been dated >50 ka, and whose phylogenetic position has been inferred from recovered genomic DNA sequences to be the sister species to the Neanderthals [2,3,8]; 4) fragmentary cranial, mandibular and dental remains from Xujiayao in North China dating in the range of ~125–60 ka, and exhibiting a unique morphology including some similarities to Neanderthals [9–11]; and 5) in Southeast Asia, *H*. *floresiensis* [12] at Liang Bua Cave, Flores, dating ~74–17 ka [13], with its similarities to Lower Pleistocene and Pliocene hominins [14,15].

The dearth of Late Pleistocene archaic fossils makes the identification of a number of the species involved in episodes of interbreeding with early anatomically modern humans (AMH) problematic. One prominent example is the occurrence of “Denisovan” DNA in the genomes of some contemporary humans in East Asia and Australo-Melanesia suggesting their Pleistocene ancestors interbred with this anatomically poorly known taxon [2,3]. While no fossils beyond Denisova Cave have been identified as belonging to this group, Denisovan mitochondrial diversity combined with the observed strong geographic patterning of Denisovan DNA in living people imply they were formerly widespread with a range extending perhaps into Southeast Asia [2,3,16–18].

Additionally, genomic research has shown that Neanderthals contributed more DNA to contemporary East Asians than Europeans indicating that multiple episodes of interbreeding must have occurred under a model of complex admixture and demographic scenarios across a wide geographic area [3,7,19]. Yet, there is presently no direct evidence for their occupation of Asia anywhere east of the Altai Mountains [8,17]. While some East Asian Middle Pleistocene remains like those from Maba in South China might show affinities to Neanderthals [20], they are probably too old to be related to the “classic” Neanderthals [21]. Yet, other interpretations of the morphology of Chinese Middle Pleistocene fossils such as from Dali have proposed a role for gene flow between archaic hominins and AMH in the emergence of recent East Asian populations [22].

Pinpointing the extinction date for archaic hominins is also important for understanding the adaptive and demographic responses of these species to the arrival of AMH beyond rare interbreeding events [1–4,8,19]. As hominins fit the definition of megafauna [23], the circumstances surrounding their extinction also potentially adds to understanding of the impacts AMH had on the environment during the Late Pleistocene and their potential role in the demise of a wide range of large-bodied mammal species [23].

Several of the current authors have previously reported the occurrence of hominin cranial remains from two sites in Southwest China (Maludong and Longlin or Laomaocao Cave) that combine archaic hominin and AMH traits [24,25]. In the case of the unusual cranium from Longlin Cave [24], it was recently concluded that its mosaic morphology probably results from hybridisation between AMH and an unknown archaic species, perhaps even occurring during the early Holocene [26]. A similar explanation might also apply to the morphology of the Maludong cranial fossils [24,25], a hypothesis currently under investigation by us. Here we report a partial proximal femur from Maludong (Yunnan), Southwest China, found in association with these unusual cranial remains, which shows morphological and phylogenetic affinities to archaic hominins. The Maludong hominins were all recovered from sediments dated with AMS ^14^C of charcoal to between about 14,310±340 cal. yr BP and 13,590±160 cal. yr BP [24,25]. Thus, the Maludong femur is the youngest fossil with archaic morphology found to date and potentially extends the overlap of AMH and archaic hominins in East Asia to >50 ka.

## Materials and Methods

Measurements for a sample of Pleistocene femora were obtained from the literature [27–29]. Comparative fossils were grouped according to chronology, in the case of Lower Pleistocene *Homo* (LPHO) and Middle Pleistocene *Homo* (MPHO); an informal grouping that represents a taxonomic unit, in the case of the Neanderthals (*H*. *neanderthalensis*, herein NEAN); and two samples of AMH: Middle Pleistocene Modern Humans (MPMH) and Early Upper-Late Upper Palaeolithic humans (EULU). A list of the specimens included in these samples is provided in S1 Table. The only specimen studied directly in the present research was MLDG 1678, which is stored at the Mengzi Institute of Cultural Relics, Mengzi, Yunnan Province, China. Permission to study it was provided by the Yunnan Provincial Culture Bureau in accordance with a memorandum of understanding between the Bureau and the University of New South Wales Australia, and by co-author Zhende Bao, Director of the Mengzi Institute of Cultural Relics, who recovered the femur during excavations in 1989.

**Fig 1:**
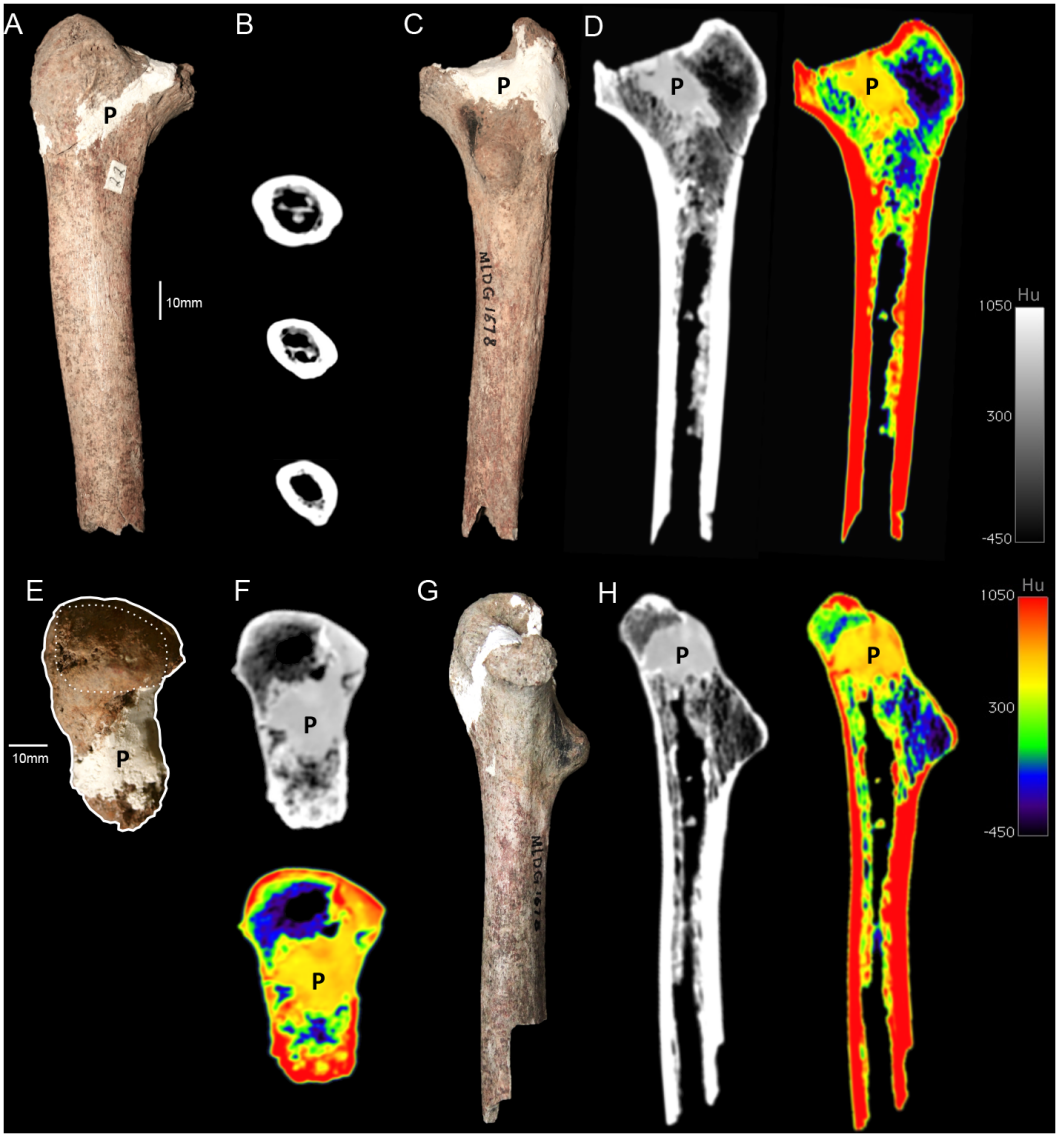
Femur MLDG 1678: (A) Anterior view. (B) CT-scan slices at subtrochanteric, approximate half-way and mid-shaft levels. (C) Posterior view. (D) CT-scan slice at approximately mid-coronal plane, grayscale (left) and colour density map (right). (E) Superior view highlighting the overall outline and superior surface of the greater trochanter (anterior at left, lateral at top). (F) CT-scan transverse slices at approximate mid-neck level, grayscale (left) and colour density map (right). (G) Medial view. (H) CT-scan slices in approximate mid-plane, grayscale (left) and colour density map (right). (P = plaster added in 1989 during restoration).

CT-scans were made of MLDG 1678 using a GE LightSpeed VCT9 scanner, with helical rotation, 0.625 mm slice thickness, KVP 140 kV and current 400 mA, at the Honghe Prefecture First Hospital during July 2012. The specimen was aligned to the relative AP and ML axes for scanning. The external and internal shaft had already been freed of matrix during curation so threshold-based segmentation was not required. However, some Plaster of Paris was added during restoration and we highlight it where it is visible in Fig 1. Measurements were selected on account of their availability on MLDG 1678. Subtrochanteric (ST) measurements were taken 10 mm below the distal edge of the lesser trochanter [30,31], while the region for mid-shaft (MS) measurements was selected following identification of a pilaster [29]. All measurements of MLDG 1678 were made by D. Curnoe on CT-scan slices following standard definitions [32] and were subsequently checked on the original specimen. Because the head is missing, neck-shaft angle was estimated using four approaches: 1) a goniometer on the original specimen; 2) a goniometer on a hardcopy (printed) photograph of the specimen; 3) the angle tool on a digital photo using the program *ImageJ*; and 4) the angle tool on a CT-scan slice at the approximate mid-coronal plane using the program *OsiriX* v6.5. We measured anteroposterior (AP) and mediolateral (ML) external diameters and cortical thickness dimensions on CT-scan slices using *OsiriX* v6.5. Total area (TA) and cortical area (CA) at the ST and MS regions were calculated using the “EEM_Macro” provided by Christopher Ruff (downloaded from: www.hopkinsmedicine.org/fae/cbr.htm).

Body mass for MLDG 1678 was estimated using the product of the ST AP and ST ML diameters and formulae from Ref [33]. Body mass was originally estimated for the comparative femora in S1 Table using head diameters [28], but these values may not be directly comparable to our estimate for MLDG 1678. Therefore, we reconstructed body mass again for the femora in our comparative samples using the same method as employed for MLDG 1678 and published AP and ML values. Size-adjustment was also undertaken on MS areas using reconstructed body mass, following the recommendations and method of Ref [34].

In scatterplots, we compared MLDG 1678 with the median, 95% confidence interval (CI) for the median and 1.5 x the interquartile range (1.5IQR) for each comparative sample. Sample 95% CIs for the median were calculated using the formula $m\pm 1.58*\frac{IQR}{\sqrt{n}}$ [35]. Doing so allowed us to visually assess for possible significance of median differences among samples [35]. We supplemented them with Kruskal-Wallis tests to assess overall significance combined with *post hoc* Mann-Whitney tests to assess pairs of samples. The inclusion of 1.5IQR for all samples, equivalent to 99.3% coverage (or 2.7 standard deviations) for normally distributed data [35], also allowed us to visually assess the affinities of MLDG 1678 in addition to its location relative to sample medians.

We undertook principal component analysis (PCA) and neighbour joining analysis using the out-group method (NJA) with the PAST program [36]. In multivariate studies of fossils a balance must be struck between information content (number of variables) and within-group variation of comparative samples in order to account for the various sources of intrasample variation (number of objects). Moreover, arbitrary decisions often need to be made about the variables to be employed. Here, we emphasised information content and undertook analyses that employed metric only and combined metric and discrete data. Variables were selected on account of their ability to distinguish between modern and archaic femora, as revealed through our morphological and univariate comparisons. Continuous variables were logged prior to analysis, while discrete traits (presence, absence) were scored on a binary scale. For NJA studies using only continuous variables we employed Euclidean distances, but for our mixed datasets, combining continuous and binary data, we used the Gower distance. Resampling scores were calculated using 100,000 replicates in all NJA investigations.

## Results

### Description and univariate comparison

MLDG 1678 consists of a 175 mm long proximal segment of an adult right femur broken slightly inferior to the MS region (Fig 1). It preserves the greater trochanter and a mostly complete neck. An isolated partial femoral head was also recovered from Maludong (MLDG 1717), but it is unclear whether is belongs to MLDG 1678. The biomechanical neck length is long, and we estimate that it must originally have measured ~70 mm; assuming MLDG 1717 provides a reasonable indication of the size of the head of MLDG 1678. Thus, it would have resembled LPHO, MPHO and NEAN, with their long biomechanical necks, rather than AMH (MPMH and EULU), with their short ones [37,38]. The horizontal AP diameter is ~27 mm, a value that would sit comfortably within the range of both archaic hominins and AMH [39].

Anteriorly, although the intertrochanteric line is somewhat obscured by plaster, owing to breakage in this region, it must have been faint. Buttressing of the proximal medial diaphysis can be seen in anterior, posterior and medial views, and is pronounced (Fig 1). The greater trochanter is a large [ML broad and superoinferiorly (SI) tall] structure that bulges beyond the lateral wall of the diaphysis. The superior surface of the neck is partly missing along its medial course, and what remains of it has been partially obscured by plaster during restoration of the specimen (Fig 1). Posteriorly, the proximal diaphysis is dominated by a massive lesser trochanter, situated on the approximate mid-plane of the shaft, and projecting slightly medially (Fig 1). CT-scan slices also show the massive size of this feature, which is composed almost entirely of cancellous bone (Fig 1). In AMH, the lesser trochanter most frequently faces strongly medially. While anteversion of the femur resulting in a more posteriorly located lesser trochanter is seen in recent humans [40], the combination of its posterior orientation and massive size in MLDG 1678 would be rare (if present at all) in AMH.

The pectineal line is a crest-like buttress running SI along the median plane of the posterior diaphysis from the base of the lesser trochanter inferiorly (Fig 1). The hypotrochanteric fossa is extensive SI and ML, merging across a wide area with the gluteal crest (S1 Fig), rather than being a distinct and narrow fossa that distinguishes the lateral wall of the femur from the weakly developed proximo-lateral buttress, as is typically seen when this feature is developed in AMH femora. It closely resembles the condition seen in NEAN in its morphology, which is probably autapomorphic for this group [41]. The intertrochanteric crest is mostly missing (not preserved), and is moderately developed inferiorly. The quadrate tubercle is not preserved.

**Fig 2:**
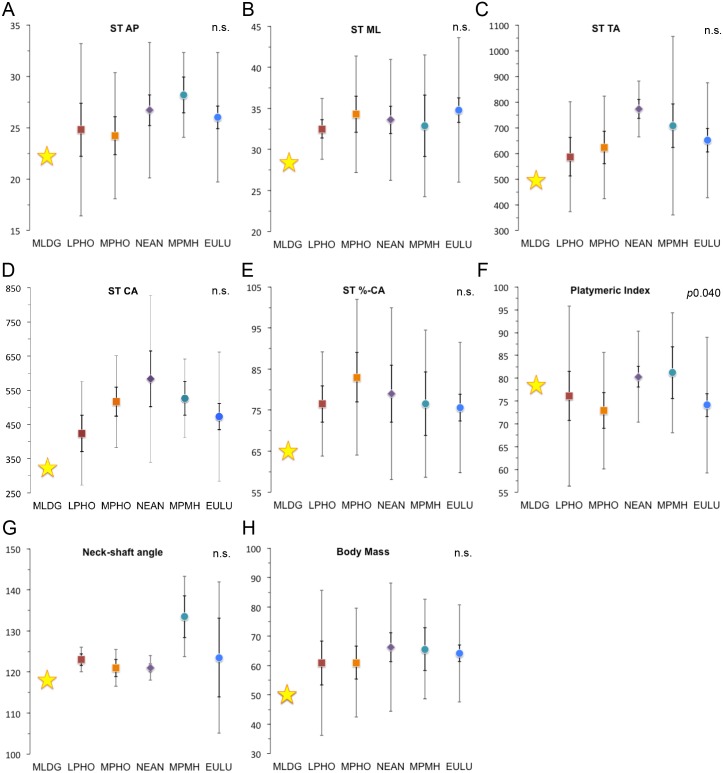
Scatterplots comparing sample medians for subtrochanteric (ST) variables, neck-shaft angle and reconstructed body mass: (A) Anteroposterior (AP) diameter (mm). (B) Mediolateral (ML) diameter (mm). (C) Total area (TA: mm^2^). (D) Cortical area (CA: mm^2^). (E) %-Cortical area (%-CA). (F) Platymeric index (%). (G) Neck-shaft angle (°). (H) Reconstructed body mass (kg). (Error bars = 95% confidence interval of median [dark] and 1.5 x interquartile range [light]; Abbreviations: LPHO = Lower Pleistocene *Homo*; MPHO = Middle Pleistocene *Homo*; NEAN = Neanderthals; MPMH = Middle Pleistocene Modern Humans; EULU = Early Upper-Late Upper Palaeolithic humans [see S1 Table]).

**Fig 3:**
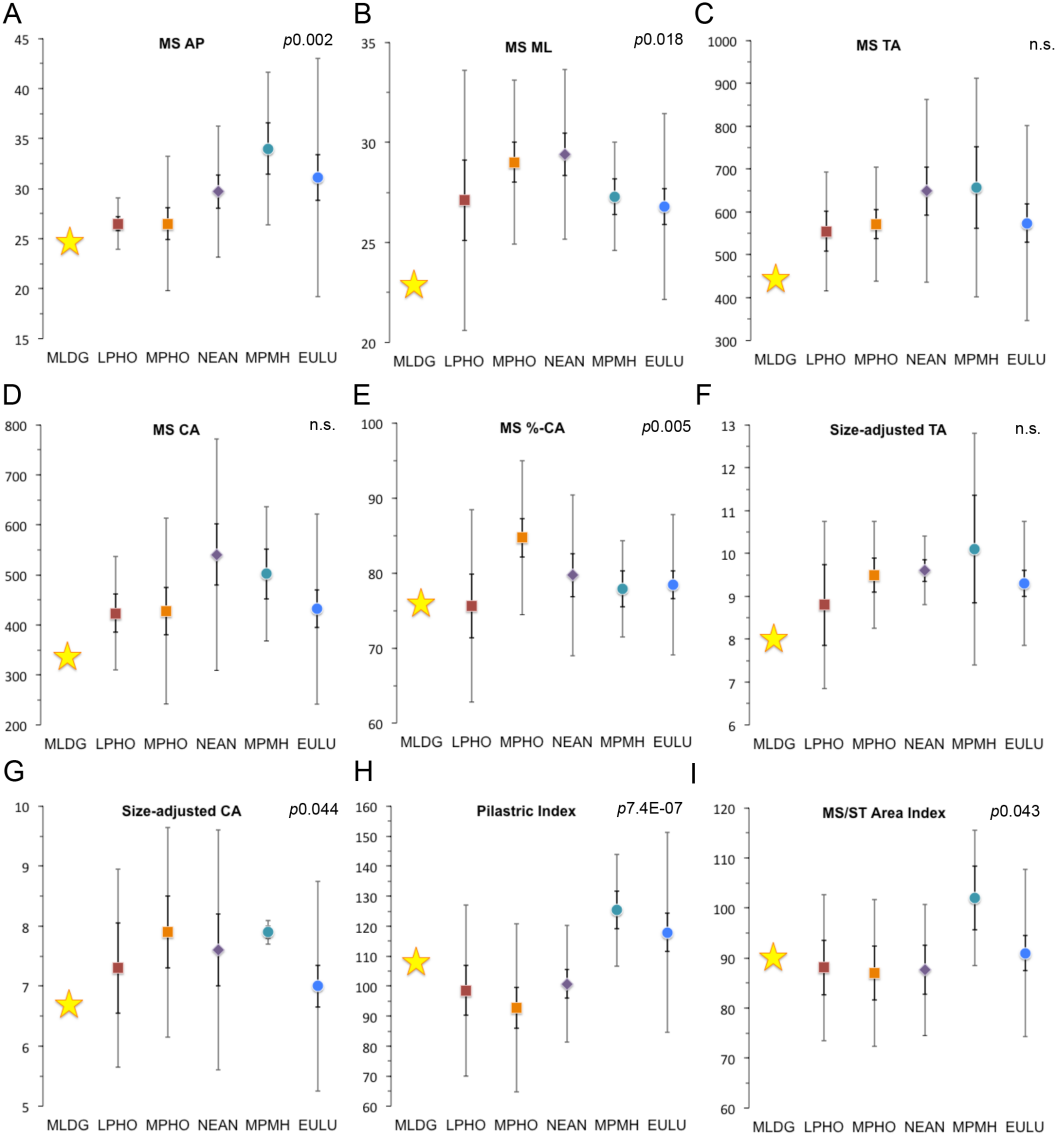
Scatterplots comparing sample medians for mid-shaft (MS) variables, size-adjusted variables and mid-shaft/subtrochanteric area index: (A) Anteroposterior (AP) diameter (mm). (B) Mediolateral (ML) diameter (mm). (C) Total area (TA: mm^2^). (D) Cortical area (CA: mm^2^). (E) %-Cortical area (%-CA). (F) Size-adjusted total area. (G) Size-adjusted cortical area. (H) Pilastric index (%). (I) Mid-shaft/subtrochanteric (MS/ST) area index (%). (Error bars = 95% confidence interval of median [dark] and 1.5 x interquartile range [light]; Abbreviations: LPHO = Lower Pleistocene *Homo*; MPHO = Middle Pleistocene *Homo*; NEAN = Neanderthals; MPMH = Middle Pleistocene Modern Humans; EULU = Early Upper-Late Upper Palaeolithic humans [see S1 Table]).

In Table 1 we present metric data for MLDG 1678. Values for the Maludong femur are compared with summary statistics for comparative samples in Figs 2 and 3. At the ST level, the AP diameter failed to show significant median differences among groups (Fig 2A). The value for MLDG 1678 is small (22.2 mm), being closest to the LPHO median, but sits within 1.5IQR of all samples except MPMH (Fig 2A). The ML diameter was also characterised by an absence of significant median differences (Fig 2B). MLDG 1678 possesses a small ML diameter (28.3 mm), again most similar to the LPHO median, but outside of the small 1.5IQR of this sample, and within 1.5IQR of all others (Fig 2B). TA at the ST level lacked significance when a Kruskal-Wallis test was performed, but *post hoc* Mann-Whitney tests revealed a significant difference between NEAN and EULU medians (*p*0.038). TA for MLDG 1678 (493.4 mm^2^) is unsurprisingly small, and once again is most similar to the LPHO median. However, its value sits with 1.5IQR of all samples except NEAN (Fig 2C). Median differences for CA also lacked significance with a Kruskal-Wallis test, but *post hoc* Mann-Whitney tests revealed a significant difference between LPHO and NEAN (*p*0.019), and NEAN and EULU (*p*0.032). CA for the Maludong femur (322.6 mm^2^) is also very small, again being most similar to the LPHO median, and with 1.5IQR only of LPHO and EULU (Fig 2D).

**Table 1:** Metric data for the Maludong femur (MLDG 1678).

	MLDG 1678
Subtrochanteric region	
Anteroposterior diameter (mm)	22.2
Mediolateral diameter (mm)	28.3
Total area (mm^2^)	493.4
Cortical area (mm^2^)	322.6
%-Cortical area	65.4
Platymeric index (%)	78.4
Neck-shaft angle (°)	~118
Reconstructed body mass (kg)	50
Mid-shaft	
Anteroposterior diameter (mm)	24.7
Mediolateral diameter (mm)	22.9
Total area (mm^2^)	444.2
Cortical area (mm^2^)	337.0
%-Cortical area	75.9
Size-adjusted total area^1^	8.8
Size-adjusted cortical area^1^	6.8
Pilastric index (%)	107.8
Mid-shaft/ST area (%)	90.0

^1^Calculated by dividing the relevant area by reconstructed body mass, after Ref [34].

For the index %-CA (CA/TA x 100), median differences were found to be non-significant. Moreover, the %-CA for MLDG 1678 (65.4%) was most similar to the LPHO and EULU medians, but sat within 1.5IQR of all samples (Fig 2E). In terms of the platymeric index, a Kruskal-Wallis test revealed significant differences among sample medians (*p*0.040): *post hoc* comparisons revealed significant differences between MPHO and NEAN (*p*0.016), and NEAN and EULU (*p*0.014) (Fig 2F). With a high platymeric index (78.4%), the shaft of the Maludong femur is reasonably circular at the ST level, being most similar to the median for NEAN (Fig 2F). While the neck-shaft angle also lacked significance with a Kruskal-Wallis test, *post hoc* Mann-Whitney tests revealed significant median differences between MPHO and MPMH (*p*0.035), NEAN and MPMH (*p*0.008), and NEAN and EULU (*p*0.035) (Fig 2G). The neck-shaft angle of MLDG 1678 is estimated to lie within the narrow range of 116–120°, and taking the median of this (c118°), it was found to be within 1.5IQR of MPHO and EULU, and equal to the negative 1.5IQR value for NEAN (Fig 2G).

We reconstructed body mass for MLDG 1678 to be low (50 kg), a value very similar to the LPHO and MPHO medians, but within 1.5IQR of all samples; although, it is clearly small by Pleistocene AMH standards (Fig 2H). Kruskal-Wallis and *post hoc* Mann-Whitney tests failed to reveal any significant differences among sample medians.

The MS AP diameter was characterised by significant median differences (Kruskal Wallis: *p*0.002). Specifically, *post hoc* tests revealed important differences between all archaic samples and MPMH (Mann Whitney: *p*0.022–0.0067), between LPHO and MPHO (*p*0.012), and LPHO and EULU (*p*0.011) (Fig 3A). Thus, this variable serves to distinguish archaic femora from Pleistocene AMH ones. The MLDG 1678 value is small (24.7 mm) and found to be most similar to the medians of LPHO and MPHO (Fig 3A). It did, however, sit within 1.5IQR of all samples except MPMH (Fig 3A). The ML diameter was also found to show significant differences among sample medians (*p*0.018), only this time EULU was distinguishable from MPHO and NEAN (*p*0.024 & *p*0.002) (Fig 3B). The Maludong ML diameter (22.9 mm) is very small and was not especially similar to any sample median, but did lie within 1.5IQR of LPHO and EULU (Fig 3B). As at the MS region, TA lacked significance in a Kruskal-Wallis test. The small area for MLDG 1678 (444.2 mm^2^) was most similar to the LPHO median, but did sit within 1.5IQR of all samples (Fig 3C). While MS CA also lacked overall significance, *post hoc* Mann-Whitney tests revealed a significant difference between NEAN and EULU medians (*p*0.039) (Fig 3D). The Maludong femur CA is very small (337.0 mm^2^), but sat within 1.5IQR of all samples except MPMH (Fig 3D). %-CA was found to exhibit significant median differences among samples (*p*0.005), with *post hoc* tests showing this to be the case between LPHO and MPHO (*p*0.025), MPHO and NEAN (*p*0.049), MPHO and MPMH (*p*0.009), and MPHO and EULU (*p*0.001) (Fig 3E). %-CA in MLDG 1678 (75.9%) was virtually identical to the LPHO median, but it did sit within 1.5IQR of all samples (Fig 3E).

We also calculated size-adjusted TA and CA using reconstructed body mass. Size-adjusted TA lacked overall significance, but *post hoc* Mann-Whitney tests revealed a significant difference between MPMH and EULU (*p*0.016) (Fig 3F). The very small value for MLDG 1678 (8.8) was most similar to the LPHO median, although, not especially close to it. Its value did, however, sit within 1.5IQR of all samples except NEAN (Fig 3F). Significant median differences were found for size-adjusted CA (*p*0.044), with *post hoc* tests showing important differences between MPHO and EULU (*p*0.029), and MPMH and EULU (*p*0.037) (Fig 3G). The small value for the Maludong femur (6.7) was most similar to the EULU median, but it did lie within 1.5IQR of all samples except MPMH (Fig 3G).

The pilastric index was found to strongly distinguish archaic hominin from AMH femora (Kruskal Wallis: *p*7.4E-07) (Fig 3H). Mann Whitney tests showed that median differences were significant between all archaic and AMH medians: archaic versus MPMH (*p*0.001–0.0003) and archaic versus EULU (*p*0.001–2.07E-05). The Maludong femur (107.8%) was found to be intermediate between archaic and AMH medians, but within 1.5IQR of all samples (Fig 3H). Finally, we also calculated an index comparing the MS TA and ST TA (MS TA/ST TA x 100) and found median differences to be significant (Kruskal-Wallis: *p*0.043) (Fig 3I). Mann-Whitney tests revealed that MPMH typically possess significantly thicker shafts at the ST level compared with archaic femora (*p*0.033–0.023), but not EULU (Fig 3I). The MS/ST area index for MLDG 1678 (90%) was most similar to the median for EULU, but again sat within 1.5IQR of all samples (Fig 3I).

### Multivariate analyses

Although the metric values for MLDG 1678 most often showed strongest affinity to the LPHO median, the individual values for the specimen were often found to lie within 1.5IQR of most comparative samples. Thus, its affinities are difficult to resolve satisfactorily using univariate methods alone, necessitating deployment of a multivariate strategy (see Materials and Methods; S1 and S2 Appendices).

**Fig 4:**
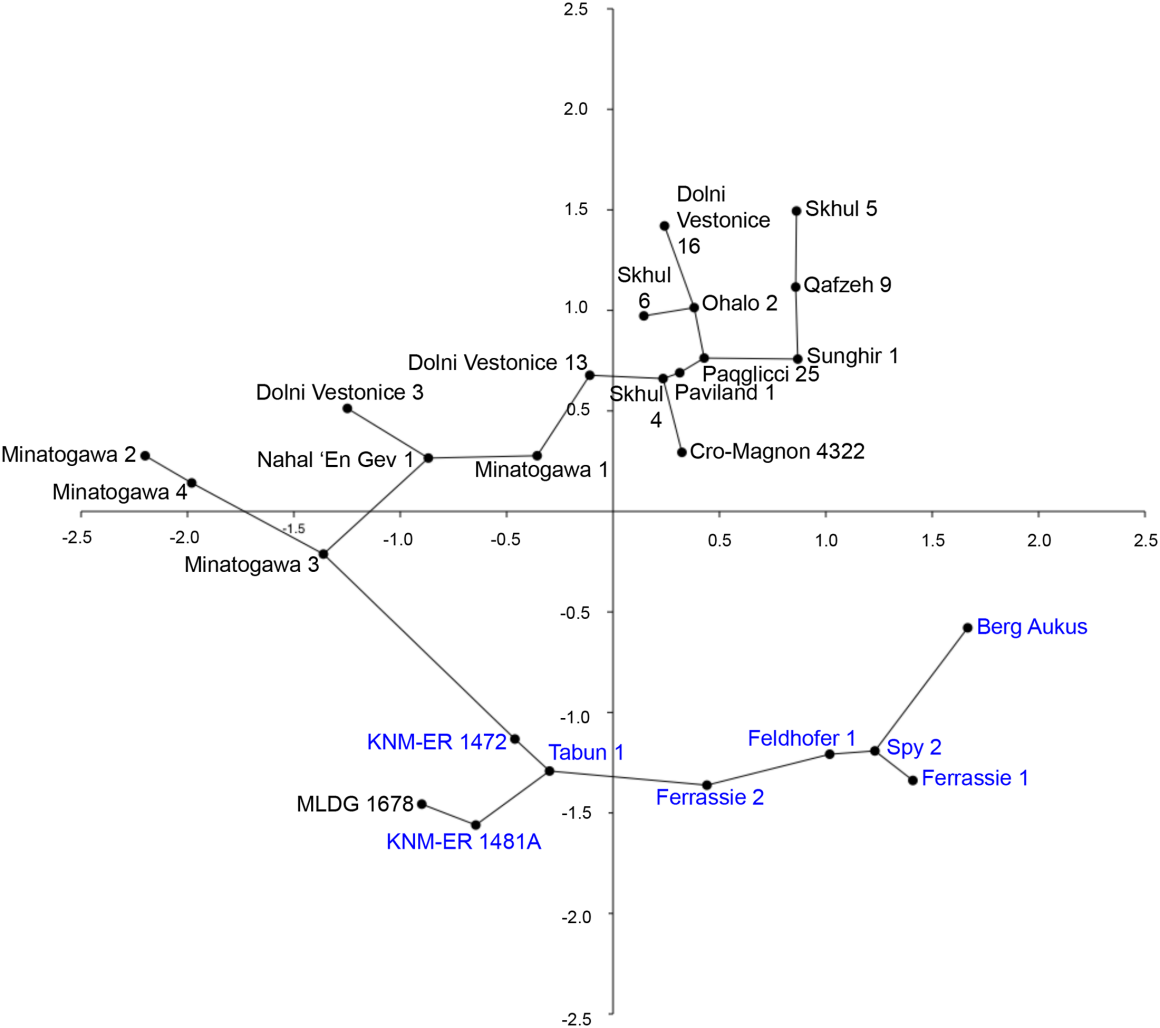
Object plot from principal component analysis of 10 continuous variables: PC1 (52.85%) versus PC2 (28.92%) (AMH femora labeled in black; archaic hominins in blue; minimal spanning tree shown).

The results of PCA employing 10 continuous variables and 26 Pleistocene femora are summarised as an object plot for principal component (PC) 1 versus PC2 in Fig 4 (see also, S2 and S3 Tables). PC1, accounting for 52.86% of total variance, showed extensive overlap of AMH (MPMH and EULU) and all archaic femora. Highest loading variables for PC1 were ST TA and ST CA. Thus, this PC highlighted strong similarities between AMH and archaic femora, especially at the ST level, which are likely to reflect common developmental responses to individual differences in femoral neck orientation and pelvic proportions [28]. In contrast, PC2 (28.92% of variance) clearly distinguished modern and archaic femora (Fig 4), the highest loading variable being MS relative cortical area (%-cortical area). This index expresses disparities between subperiosteal deposition and endosteal resorption of bone during development and is thought to be a measure of both differential developmental and aging processes as well as structural reinforcement of the diaphysis [28]. The results of PCA suggest a fundamental difference between archaic and AMH femora in this regard, as noted above in univariate comparisons, and also suggested in some previous investigations of Pleistocene femora [28]. A minimal spanning tree indicated that the shortest distance was between MLDG 1678 and KNM-ER 1481A, a specimen belonging to African early *Homo* [42,43], followed by Tabun 1, which is likely to belong in NEAN (Fig 4).

**Fig 5:**
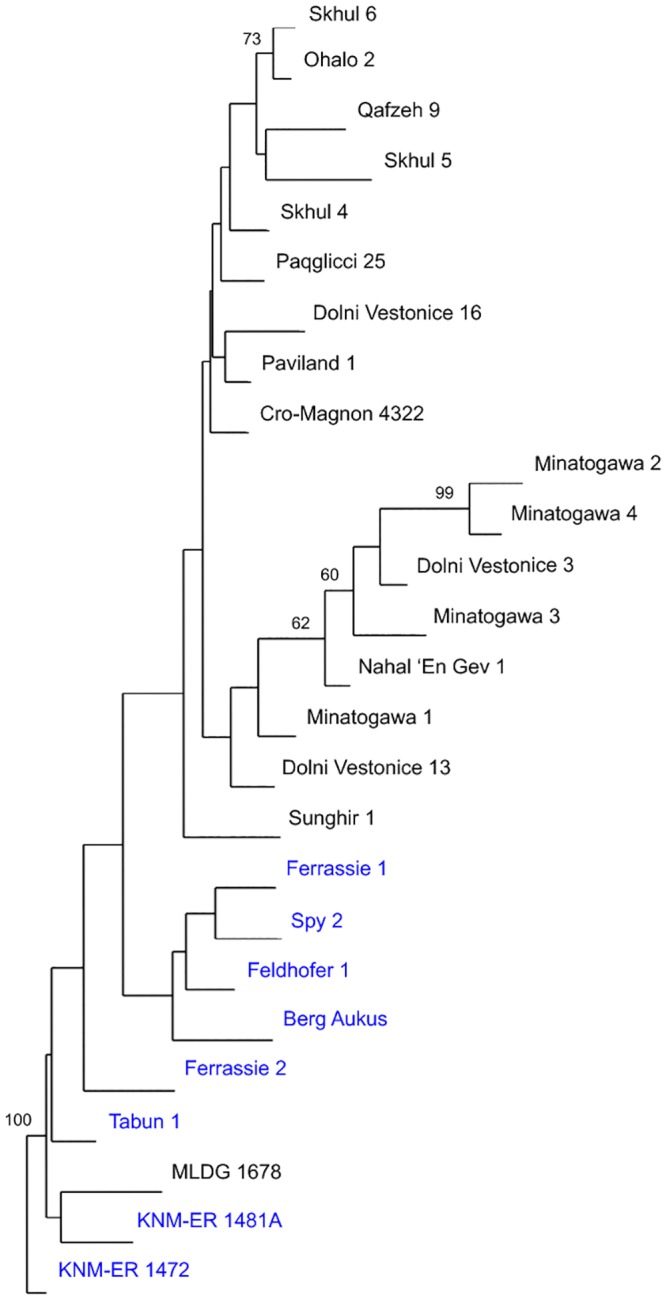
Neighbor-joining tree from an analysis of 10 continuous variables (AMH femora labeled in black; archaic hominins in blue; bootstrap scores are from 100,000 replicates with only those ≥60% shown).

We performed NJA on the same dataset employing KNM-ER 1472 (also early *Homo* [43]) as an out-group. The resulting tree also showed a clear distinction between archaic and modern human femora, although, the division did not receive strong support in bootstrap analysis (Fig 5). MLDG 1678 clustered in a branch along with KNM-ER 1481A, which was found in 100% of trees during 100,000 replicates in our resampling results (Fig 5). Thus, this tree confirmed the phenetic and phylogenetic affinities of the Maludong femur to early *Homo*.

**Fig 6:**
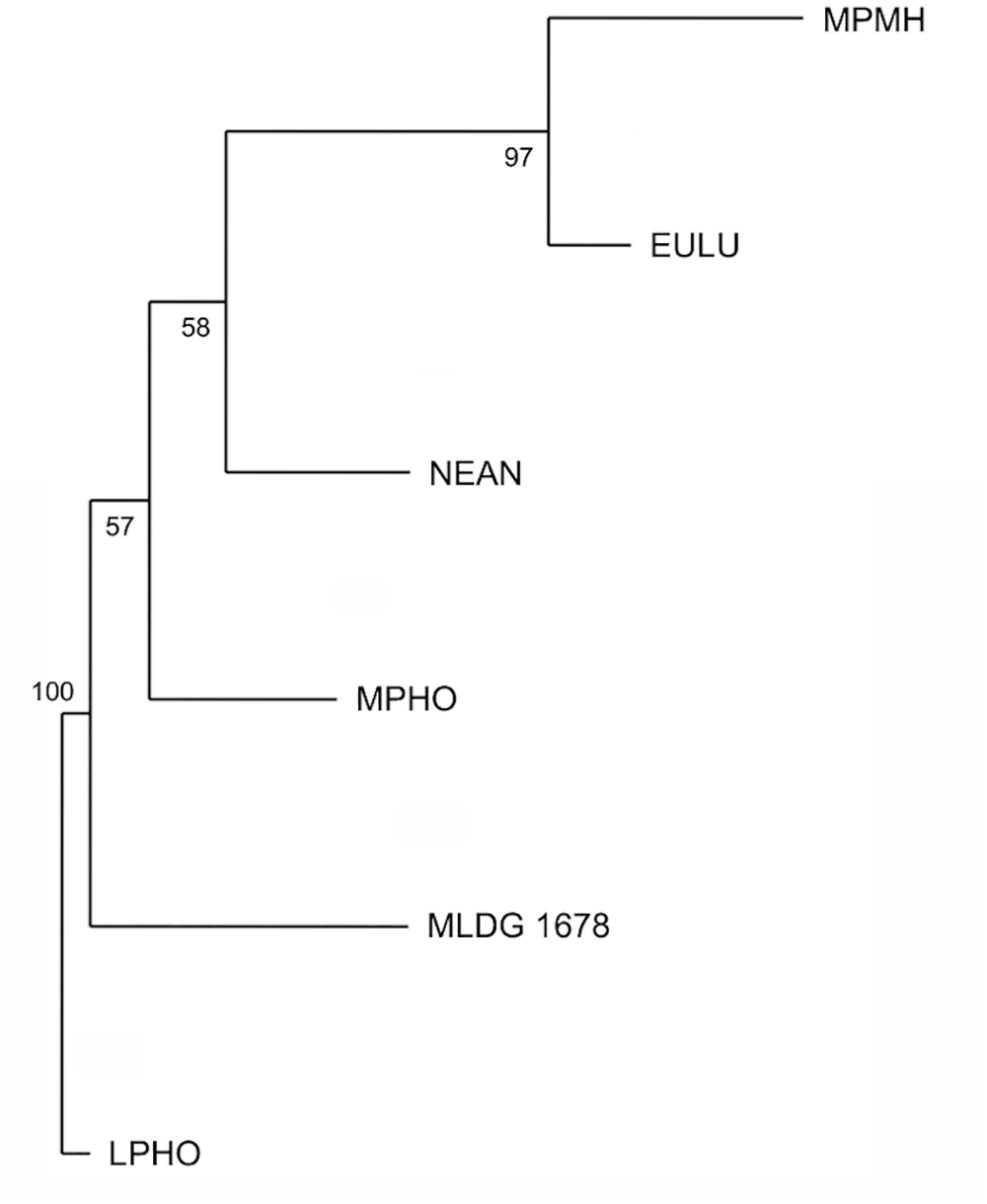
Neighbor-joining tree from an analysis of 15 variables (5 discrete and 10 continuous) using comparative sample medians as OTUs. (Bootstrap scores are from 100,000 replicates; Abbreviations: LPHO = Lower Pleistocene *Homo*; MPHO = Middle Pleistocene *Homo*; NEAN = Neanderthals; MPMH = Middle Pleistocene Modern Humans; EULU = Early Upper-Late Upper Palaeolithic humans [see S1 Table]).

The final NJA we performed included 15 variables (binary and continuous) and employed comparative sample medians (see Materials and Methods; Table 2; S2 Appendix). This time, LPHO was used as the out-group. The resulting tree placed MLDG 1678 as the most basal branch separate to a branch containing MPHO, MPMH and EULU (Fig 6). The branch containing MLDG 1678 was found in 100% of trees during 100,000 bootstrap replicates (Fig 6). The results showed the Maludong femur to be highly plesiomorphic and morphologically and phylogenetically distinct from Middle and Late Pleistocene hominins.

**Table 2:** Comparison of systematically relevant traits in MLDG 1678^1^.

	MLDG 1678	LPHO	MPHO	NEAN	MPMH	EULU
1. Biomechanical neck length	Long	Long	Long	Long	Short	Short
2. Lesser trochanter: large, posteriorly oriented	Present	Present	Variable	Absent	Absent	Absent
3. Pronounced medial buttressing	Present	Present	Present	Present	Absent	Absent
4. Marked gluteal buttress	Absent	Absent	Absent	Absent	Present	Present
5. Hypotrochanteric fossa merges with gluteal buttress	Present	Absent	Absent	Present	Absent	Absent
6. ST Total area	Small	Small	Small	Large	Moderate	Moderate
7. ST Cortical area	Very small	Small	Moderate	Large	Moderate	Moderate
8. Platymeric index	Large	Small	Small	Large	Large	Small
9. Neck-shaft angle	Low	Low	Low	Low	High	Low
10. MS AP diameter	Small	Small	Small	Moderate	Large	Moderate
11. MS ML diameter	Very small	Small	Large	Large	Small	Small
12. MS Size-adjusted cortical area	Small	Small	Large	Large	Large	Small
13. MS %-Cortical area	Low	Low	High	Low	Low	Low
14. Pillastric index	Moderate	Low	Low	Low	High	High
15. Mid-shaft/ST area	Low	Low	Low	Low	High	Low

^1^Abbreviations: LPHO = Lower Pleistocene *Homo*; MPHO = Middle Pleistocene *Homo*; NEAN = Neanderthals; MPMH = Middle Pleistocene Modern Humans; EULU = Early Upper-Late Upper Palaeolithic humans. ST = subtrochanteric region; MS = mid-shaft region; AP = anteroposterior diameter; ML = mediolateral diameter.

Sample compositions, see S1 Table.

## Discussion and Conclusions

In light of the absence of archaic hominins from mainland Eurasia younger than about 39 ka [5,11,17,20] it would be reasonable to expect MLDG 1678 to sample an Upper Palaeolithic individual, given its young geological age (i.e. ~14.0 ka). Instead, our comparisons with Pleistocene femora reveal the Maludong specimen to be morphologically distinct from AMH. Moreover, of the 15 traits summarised in Table 2, eight are shared with archaic hominins to the exclusion of Pleistocene AMH femora, and a further two are unusual in MLDG 1678 by Late Pleistocene hominin standards. The others failed to distinguish AMH from one or more archaic samples, so have little bearing directly on the affinities of MLDG 1678 (Table 2). It is striking that for the five discrete traits compared, the Maludong femur shares states only with archaic hominins, differentiating them all from AMH (Table 2). Noteworthy, overall, is its: 1) small ST TA and CA, the latter being suggestive of low resistance to axial loads [28]; 2) relatively high platymeric index, indicating its shaft to be rather circular at the ST level; 3) small MS AP and ML diameters, which could reflect relatively low levels, although, the level of buttressing seen in the Maludong femur suggests otherwise and perhaps instead results from its small body size [34]; and 4) moderate pilastric index value, associated with the presence of a weakly developed femoral pilaster, a feature often taken to distinguish AMH from archaic hominins [44] (Table 2).

Putting the results of our analyses together, MLDG 1678 shows morphological and phylogenetic affinities to archaic femora, particularly Lower Pleistocene early *Homo*. We conclude, therefore, that the Maludong femur represents an individual that probably belonged to an archaic taxon rather than AMH. Just which taxon it represents is difficult to determine, however, because of the scarcity of Late Pleistocene hominin femora from East Asia. It also remains unclear whether Late Pleistocene archaic groups from western Eurasia provide the most appropriate model for East Asian hominins during this period. In the case of AMH, though, we did include femora from East Asia, including the four from Minatogawa, and MLDG 1678 was found to be very distinct from them.

It is intriguing that such a plesiomorphic hominin could have survived at Maludong until near the end of the Pleistocene. Yet, this finding applies also to *H*. *floresiensis*, with its apparent minimum geological age only slightly older than MLDG 1678 [13]. *Homo floresiensis* has only been found on the island of Flores in eastern Indonesia though, and its occurrence has been explained by island biogeography [45,46]. Moreover, the Maludong femur is distinct from the highly unusual femora of this species, with its unique mosaic of traits including resemblances to *Australopithecus* taxa [47]. One possible explanation is that the Maludong femur samples the population presently known only from Denisova Cave in the Altai region and dubbed the “Denisovans” [2,3]. Another candidate is the presently unnamed taxon represented by the Xujiayao fossils [9–11]. However, the absence of femora from both of these groups makes these scenarios impossible to test at present. Besides, the similarities of MLDG 1678 to Lower Pleistocene hominins implies that other possibilities should be considered, such as a late surviving descendent of a Lower Pleistocene East Asian group [48] or even the Dmanisi hominins [49].

Yunnan Province is characterised by complex topography associated with Himalayan uplift and extrusion of the Indochina block resulting in vicarious biogeographic divisions [50]. It is one of 20 floristic endemic centres in China, comprising subtropical evergreen broad-leaved and sclerophyllous forests, and contains high levels of palaeoendemism [50–52]. The region around Maludong is also biogeographically on the northern edge of tropical Southeast Asia [50]. Thus, the Maludong femur might represent a relic, tropically adapted, archaic population that survived relatively late in the biogeographically complex region of Southwest China.

## Supporting Information

S1 Appendix Ten variable matrix employed in PCA and NJA of individual fossils. (DOCX)

S2 Appendix Fifteen variable matrix used for NJA with samples as OTUs. (DOCX)

S1 Fig Lateral view of MLDG 1678. (DOCX)

S1 Table Composition of comparative samples. (DOCX)

S2 Table Results of PCA. (DOCX)

S3 Table Variable loadings from PCA. (DOCX)
