# Supplementary material for: A Hominin Femur with Archaic Affinities from the Late Pleistocene of Southwest China
Source: PLoS One. 2015 Dec 17;10(12):e0143332. doi: 10.1371/journal.pone.0143332 (PMC4683062; doi:10.1371/journal.pone.0143332)
Supplement: S1 Appendix — (DOCX) [file pone.0143332.s001.docx]

**S1 Appendix**. Ten variable matrix (continuous variables) employed in PCA and NJA of individual fossils (values are logged).

|  | ST Total | ST Cortical | Neck shaft | Platymeric | MS AP | MS ML | MS Size- | MS %- | Pillastric | MS/ST |
| --- | --- | --- | --- | --- | --- | --- | --- | --- | --- | --- |
|  | area | area | angle | index | diameter | diameter | adjusted | cortical | index | area |
|  |  |  |  |  |  |  | cortical area | area |  |  |
| KNM-ER 1472 | 2.701 | 2.627 | 2.097 | 1.842 | 1.417 | 1.423 | 0.878 | 1.936 | 1.993 | 1.966 |
| MLDG 1678 | 2.693 | 2.509 | 2.072 | 1.895 | 1.393 | 1.360 | 0.833 | 1.880 | 2.033 | 1.954 |
| KNM-ER1481A | 2.723 | 2.617 | 2.090 | 1.827 | 1.352 | 1.403 | 0.811 | 1.929 | 1.949 | 1.869 |
| Berg Aukus | 2.925 | 2.872 | 2.025 | 1.866 | 1.555 | 1.498 | 0.926 | 1.947 | 2.057 | 1.978 |
| Feldhofer 1 | 2.880 | 2.774 | 2.086 | 1.917 | 1.496 | 1.450 | 0.834 | 1.888 | 2.045 | 1.935 |
| Ferrassie 1 | 2.952 | 2.822 | 2.079 | 1.916 | 1.474 | 1.505 | 0.874 | 1.894 | 1.969 | 1.906 |
| Ferrassie 2 | 2.857 | 2.676 | 2.104 | 1.940 | 1.422 | 1.471 | 0.936 | 1.894 | 1.950 | 2.001 |
| Spy 2 | 2.987 | 2.758 | 2.064 | 1.896 | 1.467 | 1.464 | 0.835 | 1.903 | 2.003 | 1.803 |
| Tabun 1 | 2.746 | 2.648 | 2.093 | 1.871 | 1.380 | 1.438 | 0.905 | 1.934 | 1.942 | 1.951 |
| Qafzeh 9 | 2.827 | 2.762 | 2.117 | 1.926 | 1.577 | 1.464 | 1.074 | 2.091 | 2.114 | 1.968 |
| Skhul 4 | 2.797 | 2.679 | 2.134 | 1.893 | 1.526 | 1.401 | 0.945 | 2.073 | 2.125 | 1.881 |
| Skhul 5 | 2.872 | 2.693 | 2.121 | 2.052 | 1.598 | 1.441 | 1.007 | 2.117 | 2.157 | 1.850 |
| Skhul 6 | 2.771 | 2.660 | 2.134 | 1.935 | 1.537 | 1.431 | 1.006 | 2.100 | 2.105 | 1.911 |
| Cro-Magnon 4322 | 2.820 | 2.696 | 2.097 | 1.857 | 1.505 | 1.430 | 0.914 | 2.067 | 2.075 | 1.858 |
| Dolni Vestonice 3 | 2.644 | 2.531 | 2.114 | 1.822 | 1.418 | 1.367 | 0.935 | 2.123 | 2.051 | 1.841 |
| Dolni Vestonice 13 | 2.768 | 2.679 | 2.079 | 1.825 | 1.470 | 1.433 | 0.956 | 2.143 | 2.037 | 1.835 |
| Dolni Vestonice 16 | 2.833 | 2.637 | 2.097 | 1.907 | 1.556 | 1.425 | 0.948 | 2.170 | 2.131 | 1.793 |
| Minatogawa 1 | 2.737 | 2.657 | 2.086 | 1.865 | 1.423 | 1.423 | 0.986 | 2.095 | 2.000 | 1.866 |
| Minatogawa 2 | 2.551 | 2.430 | 2.124 | 1.857 | 1.322 | 1.342 | 0.964 | 2.134 | 1.980 | 1.858 |
| Minatogawa 3 | 2.594 | 2.542 | 2.146 | 1.864 | 1.380 | 1.371 | 0.980 | 2.030 | 2.009 | 1.968 |
| Minatogawa 4 | 2.563 | 2.487 | 2.121 | 1.852 | 1.322 | 1.362 | 0.912 | 2.142 | 1.960 | 1.809 |
| Nahal 'En-Gev 1 | 2.677 | 2.568 | 2.121 | 1.895 | 1.423 | 1.365 | 0.970 | 2.067 | 2.058 | 1.914 |
| Ohalo 2 | 2.801 | 2.682 | 2.117 | 1.938 | 1.549 | 1.431 | 1.019 | 2.093 | 2.118 | 1.914 |
| Paglicci 25 | 2.789 | 2.739 | 2.068 | 1.871 | 1.531 | 1.431 | 0.967 | 2.106 | 2.100 | 1.894 |
| Paviland 1 | 2.833 | 2.683 | 2.083 | 1.873 | 1.509 | 1.438 | 0.942 | 2.116 | 2.071 | 1.835 |
| Sunghir 1 | 2.899 | 2.739 | 2.090 | 1.837 | 1.539 | 1.529 | 1.019 | 2.125 | 2.010 | 1.872 |
