# Supplementary material for: A Hominin Femur with Archaic Affinities from the Late Pleistocene of Southwest China
Source: PLoS One. 2015 Dec 17;10(12):e0143332. doi: 10.1371/journal.pone.0143332 (PMC4683062; doi:10.1371/journal.pone.0143332)
Supplement: S2 Appendix — (DOCX) [file pone.0143332.s002.docx]

**S2 Appendix**. Fifteen variable matrix (discrete and continuous) used for NJA with samples as OTUs (continuous variables have been logged).

|  |  | Neck | Lesser | Pronounced | Marked | Hypotroch. | ST Total | ST Cortical | Neck shaft | Platymeric |
| --- | --- | --- | --- | --- | --- | --- | --- | --- | --- | --- |
|  |  | length | trochanter: | medial | gluteal | fossa | area | area | angle | index |
|  |  |  | large, post. | buttressing | buttress | merges with |  |  |  |  |
|  |  |  | directed |  |  | gluteal butt. |  |  |  |  |
| LPHO (Out-group) |  | 0 | 0 | 0 | 0 | 0 | 2.769 | 2.627 | 2.090 | 1.881 |
| MLDG 1678 |  | 0 | 0 | 0 | 0 | 1 | 2.693 | 2.509 | 2.072 | 1.894 |
| MPHO |  | 0 | 0 | 0 | 0 | 0 | 2.794 | 2.713 | 2.083 | 1.863 |
| NEAN |  | 0 | 1 | 0 | 0 | 1 | 2.888 | 2.766 | 2.083 | 1.905 |
| MPMH |  | 1 | 1 | 1 | 1 | 0 | 2.850 | 2.721 | 2.125 | 1.910 |
| EULU |  | 1 | 1 | 1 | 1 | 0 | 2.814 | 2.675 | 2.092 | 1.870 |

**S2 Appendix**. *Continued*.

|  |  | MS AP | MS ML | MS Size- | MS %- | Pillastric | MS/ST |
| --- | --- | --- | --- | --- | --- | --- | --- |
|  |  | diameter | diameter | adjusted | cortical | index | area |
|  |  |  |  | cortical area | area |  |  |
| LPHO (Out-group) |  | 1.423 | 1.433 | 0.863 | 1.879 | 1.993 | 1.945 |
| MLDG 1678 |  | 1.393 | 1.360 | 0.826 | 1.880 | 2.033 | 1.954 |
| MPHO |  | 1.423 | 1.462 | 0.898 | 1.928 | 1.967 | 1.940 |
| NEAN |  | 1.473 | 1.468 | 0.881 | 1.901 | 2.003 | 1.943 |
| MPMH |  | 1.531 | 1.436 | 0.898 | 1.892 | 2.098 | 2.009 |
| EULU |  | 1.493 | 1.428 | 0.845 | 1.894 | 2.072 | 1.959 |
