# Supplementary material for: A Hominin Femur with Archaic Affinities from the Late Pleistocene of Southwest China
Source: PLoS One. 2015 Dec 17;10(12):e0143332. doi: 10.1371/journal.pone.0143332 (PMC4683062; doi:10.1371/journal.pone.0143332)
Supplement: S1 Fig — (DOCX) [file pone.0143332.s003.docx]

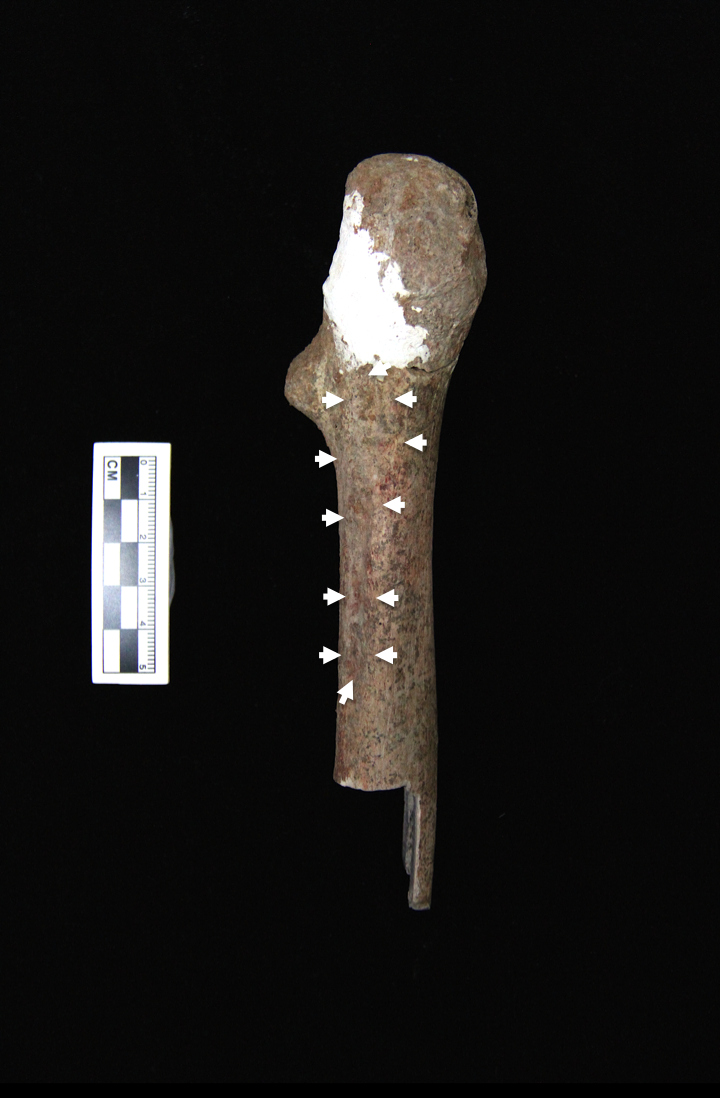


**S1 Fig.** Lateral view of MLDG 1678. Arrows indicate the expansive hypotrochantric fossa that merges with the gluteal crest.
