# Supplementary material for: A Hominin Femur with Archaic Affinities from the Late Pleistocene of Southwest China
Source: PLoS One. 2015 Dec 17;10(12):e0143332. doi: 10.1371/journal.pone.0143332 (PMC4683062; doi:10.1371/journal.pone.0143332)
Supplement: S1 Table — (DOCX) [file pone.0143332.s004.docx]

**S1 Table**. Composition of comparative samples.

| **Lower Pleistocene *Homo* (LPHO)** |  | | |
| --- | --- | --- | --- |
| KNM-ER 736 | OH 34 | | |
| KNM-ER 737 | OH 62 | | |
| KNM-ER 803 | Trinil 1 | | |
| KNM-ER 999 | Trinil 2 | | |
| KNM-ER 1472 | Trinil 3 | | |
| KNM-ER 1475 | Trinil 4 | | |
| KNM-ER 1481A | Dmanisi D4167 | | |
| KNM-ER 3728 |  | | |
|  |  | | |
| **Middle Pleistocene *Homo* (MPHO)** |  | | |
| Aïn Maarouf 1 | Kresna 11 | | |
| Arago 48 | Mammolo 1 | | |
| Berg Aukus 1 | OH 28 | | |
| Broken Hill E689 | Sedia-del-Diavolo 1 | | |
| Broken Hill E690 | Tabun E1 | | |
| Broken Hill E793 | Zhoukoudian 1 | | |
| Castel del Guido 1 | Zhoukoudian 2 | | |
| La Chaise-BD 5 | Zhoukoudian 4 | | |
| Ehringsdorf 5 | Zhoukoudian 5 | | |
| Gesher-B.-Y. 1 | Zhoukoudian 6 | | |
| Gesher-B.-Y. 2 |  | | |
|  |  | | |
| **Neanderthals (NEAN)** |  | | |
| Amud 1 | Quina 38 | | |
| Chapelle-aux-Saints 1 | Rochers-de-V. 1 | | |
| Feldhofer 1 | Saint Césaire 1 | | |
| Ferrassie 1 | Santa Croce 1 | | |
| Ferrassie 2 | Shanidar 1 | | |
| Fond-de-Forêt 1 | Shanidar 4 | | |
| Hortus 34 | Shanidar 5 | | |
| Krapina 213 | Shanidar 6 | | |
| Krapina 214 | Spy 2 | | |
| Krapina 257.32 | Stadelhöle 1 | | |
| Krapina 257.33 | Tabun 1 | | |
| Palomas 52 | Tabun 3 | | |
| Palomas 96 | Zafarraya 1 | | |
| Quina 5 |  | | |
|  |  | | |
| **Middle Pleistocene Modern Humans (MPMH)** |  | | |
| Qafzeh 3 | Skhul 6 | | |
| Qafzeh 8 | Skhul 7 | | |
| Qafzeh 9 | Skhul 9 | | |
| Skhul 3 | Skhul ‘7’ | | |
| Skhul 4 | Skhul ‘9’ | | |
| Skhul 5 |  | | |
|  |  | | |
| **Early Upper-Late Upper Palaeolithic humans (EULU)** | |  |  |
| Arene Candide 1 | Mladec 28 | | |
| Barma Grande 1 | Nahal ´En-Gev 1 | | |
| Barma Grande 2 | Ohalo 2 | | |
| Barma Grande 6 | Paglicci 25 | | |
| Caviglione 1 | Paviland 1 | | |
| Cro Magnon 1 | Pavlov 1 | | |
| Cro Magnon 4323A | Predmostí 3 | | |
| Cro Magnon 4322 | Predmostí 4 | | |
| Cro Magnon 4324 | Predmostí 9 | | |
| Dolní Vestonice 3 | Predmostí 10 | | |
| Dolní Vestonice 13 | Predmostí 14 | | |
| Dolní Vestonice 14 | Rochette 2 | | |
| Dolní Vestonice 16 | Sunghir 1 | | |
| Dolní Vestonice 35 | Sunghir 4 | | |
| Grotte-de-Enfants 4 | Tianyuan 1 | | |
| Grotte-des-Enfants 5 | Veneri 1 | | |
| Minatogawa 1 | Veneri 2 | | |
| Minatogawa 2 | Willendorf 1 | | |
| Minatogawa 3 | Zhoukoudian UC 67 | | |
| Minatogawa 4 | Zhoukoudian UC 68 | | |
| Mladec 27 |  | | |
