# Supplementary material for: A Hominin Femur with Archaic Affinities from the Late Pleistocene of Southwest China
Source: PLoS One. 2015 Dec 17;10(12):e0143332. doi: 10.1371/journal.pone.0143332 (PMC4683062; doi:10.1371/journal.pone.0143332)
Supplement: S2 Table — (DOCX) [file pone.0143332.s005.docx]

**S2 Table**. Results of PCA.

| PC | Eigenvalue | % Variance | % Cumulative |
| --- | --- | --- | --- |
|  |  |  | variance |
| 1 | 0.030 | 52.86 | 52.86 |
| 2 | 0.016 | 28.92 | 81.78 |
| 3 | 0.005 | 8.17 | 89.95 |
| 4 | 0.003 | 5.47 | 95.43 |
