# Supplementary material for: A Hominin Femur with Archaic Affinities from the Late Pleistocene of Southwest China
Source: PLoS One. 2015 Dec 17;10(12):e0143332. doi: 10.1371/journal.pone.0143332 (PMC4683062; doi:10.1371/journal.pone.0143332)
Supplement: S3 Table — (DOCX) [file pone.0143332.s006.docx]

**S3 Table**. Variable loadings for first four PCs from PCA.

|  | PC 1 | PC 2 | PC 3 | PC 4 |
| --- | --- | --- | --- | --- |
| ST total area | 0.640 | -0.054 | -0.252 | -0.133 |
| ST cortical area | 0.576 | -0.033 | -0.041 | 0.356 |
| Neck shaft angle | -0.078 | 0.082 | 0.143 | -0.066 |
| Platymeric index | 0.122 | 0.085 | 0.330 | -0.462 |
| MS AP diameter | 0.367 | 0.346 | 0.154 | -0.172 |
| MS ML diameter | 0.241 | -0.024 | -0.052 | 0.297 |
| MS size-adjusted cortical area | 0.014 | 0.415 | 0.369 | 0.415 |
| MS %-cortical area | -0.160 | 0.716 | -0.276 | 0.238 |
| Pillastric index | 0.126 | 0.372 | 0.206 | -0.468 |
| MS/ST area | 0.050 | -0.196 | 0.723 | 0.268 |
